# Brachial cuff measurements for fluid responsiveness prediction in the critically ill

**DOI:** 10.1186/cc9493

**Published:** 2011-03-11

**Authors:** K Lakhal, S Ehrmann, D Benzekri-Lefèvre, I Runge, A Legras, E Mercier, PF Dequin, M Wolff, B Régnier, T Boulain

**Affiliations:** 1 CHU, Montpellier, France; 2 CHRU, Tours, France; 3 CHR, Orléans, France; 4 Hopital Bichat-C.Bernard, Paris, France

## Introduction

The passive leg raising maneuver (PLR) with concomitant measurement of invasive arterial pressure (AP) or cardiac output (CO) changes are used to test volume responsiveness. The initial hemodynamic evaluation of shocked patients often relies on the sole non-invasive measurement of AP. We assessed the performance of PLR-induced changes in oscillometric measurements of systolic, mean and pulse AP (ΔplrSAP, ΔplrMAP and ΔplrPP).

## Methods

CO and AP measurements were performed before/during PLR and then after 500 ml volume expansion.

## Results

**Figure 1:**
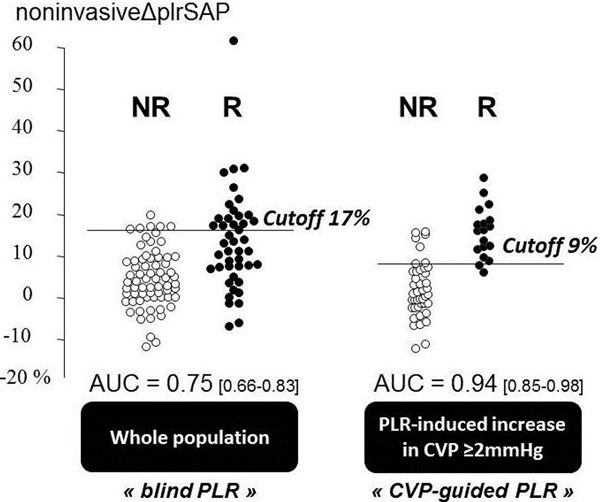


In 112 patients, the area under the ROC curve (AUC) of ΔplrSAP was 0.75 (0.66 to 0.83). When ΔplrSAP was >17%, the positive likelihood ratio (LHR) was 26 (18 to 38). Non-invasive ΔplrPP and non-invasive ΔplrMAP were associated with an AUC of 0.70 (0.61 to 0.79) and 0.69 (0.59 to 0.77), respectively. If PLR induced change in central venous pressure (CVP) it was ≥2 mmHg (*n *= 60), suggesting that PLR actually changed the cardiac preload, AUC of ΔplrSAP was 0.90 (0.80 to 0.97). In these patients, ΔplrSAP >9% was associated with a positive and negative LHR of 5.7 (4.6 to 6.8) and 0.07 (0.009 to 0.5), respectively. See Figure 1.

## Conclusions

Regardless of CVP (blind PLR), ΔplrSAP >17% reliably identified responders. CVP-guided PLR allowed ΔplrSAP to perform better in the case of sufficient change in preload during PLR.

